# JC2-11, a benzylideneacetophenone derivative, attenuates inflammasome activation

**DOI:** 10.1038/s41598-022-27129-3

**Published:** 2022-12-28

**Authors:** Gilyoung Lee, Huijeong Ahn, Jang-Hyuk Yun, Jeongho Park, Eunsong Lee, Seikwan Oh, Geun-Shik Lee

**Affiliations:** 1 College of Veterinary Medicine and Institute of Veterinary Science, Kangwon National University, Chuncheon, Gangwon 24341 Republic of Korea; 2 Department of Molecular Medicine, School of Medicine, Ewha Womans University, Gangseo-gu, Seoul, 07804 Republic of Korea

**Keywords:** Inflammation, Innate immunity

## Abstract

Dysregulation of inflammasome activation induces chronic and excess inflammation resulting in several disorders, such as metabolic disorders and cancers. Thus, screening for its regulator derived from natural materials has been conducted progressively. JC2-11 (JC) was designed to enhance the antioxidant activity based on a chalcone, which is abundant in edible plants and a precursor of flavonoids. This study examined the effects of JC on inflammasome activation in human and murine macrophages. JC inhibited the secretion of interleukin (IL)-1β and lactate dehydrogenases, and the cleavage of caspase-1 and gasdermin D in response to the tested activators (i.e., NLRP3, NLRC4, AIM2, and non-canonical inflammasome triggers). In addition, JC attenuated IL-1β secretion from lipopolysaccharide (LPS)-injected mice, an inflammasome-mediating disease model. Mechanistically, JC blocked the expression of the inflammasome components during the priming step of the inflammasome, and interrupted the production of mitochondrial reactive oxygen species. In addition, JC inhibited the activity of caspase-1. In conclusion, JC may be a candidate pan-inflammasome inhibitor.

## Introduction

Inflammasomes, intracellular multi-protein complexes, are formed by recognizing the cytosolic danger signals resulting in the secretion of pro-inflammatory cytokines (e.g., interleukin [IL]-1β and -18) and the induction of inflammatory cell death (i.e., pyroptosis) in innate immune and epithelial cells^1,2^. Inflammasomes consist of a sensor protein, an adaptor protein (apoptosis-associated speck-like protein containing a caspase recruitment domain [ASC]), and caspase-1. Its name is determined according to the sensor protein, such as nucleotide-binding oligomerization domain-like receptor (NLR) pyrin domain-containing protein 3 (NLRP3), NLR caspase activation, and recruitment domain-containing protein 4 (NLRC4), and absent in melanoma 2 (AIM2) inflammasomes^1,2^. NLRP3 inflammasome is assembled by a common intracellular signal (e.g., potassium efflux and mitochondrial reactive oxygen species [ROS]) that is induced by microbial or self-derived danger signals, such as adenosine triphosphate (ATP), bacterial toxin (e.g., nigericin), uric acid crystals (MSU), amyloid-β fibrils, and cholesterol crystals. On the other hand, NLRC4 and AIM2 interact directly with cytosolic pathogen components (e.g., flagellin and dsDNA) to form inflammasome^1,2^. The inflammasome assembly leads to self-activate caspase-1, which induces proteolytic cleavage of the pro-forms of IL-1β, IL-18, and gasdermin D (GSDMD). The cleavage GSDMD forms a pore on the cytoplasmic membrane, and cytosolic components, such as cytokines, inflammasome components, and lactate dehydrogenases (LDH), are emitted through the pores^1,3^. This pyroptosis interrupts the infection of the host cells by microbes^3,4^. Unlike the above canonical inflammasomes, murine caspase-11 (caspase-4/5 for humans) interacts with cytosolic lipopolysaccharide (LPS) and then induces pyroptosis by GSDMD cleavage and activates NLRP3 inflammasome indirectly, making it a non-canonical inflammasome^5–7^. For successful inflammasome activation, the inflammasome components and substrates need to be upregulated^1,8^. This priming step is achieved mainly through toll-like receptor (TLR)-nuclear factor (NF)-κB signaling^1,8^. Although inflammasome activation contributes to homeostatic inflammation (e.g., eliminating harmful molecules and repairing damaged tissues), excessive and chronic inflammasome activation causes several metabolic and degenerative diseases, such as Alzheimer’s diseases, type 2 diabetes mellitus, arteriosclerosis, gout, and carcinogenesis^9^. Thus, studies of a natural material capable of regulating the inflammasome activation have been conducted^5,10–14^. Moreover, several studies have examined flavonoids, including chalcones^15–20^.

**Figure 1:**
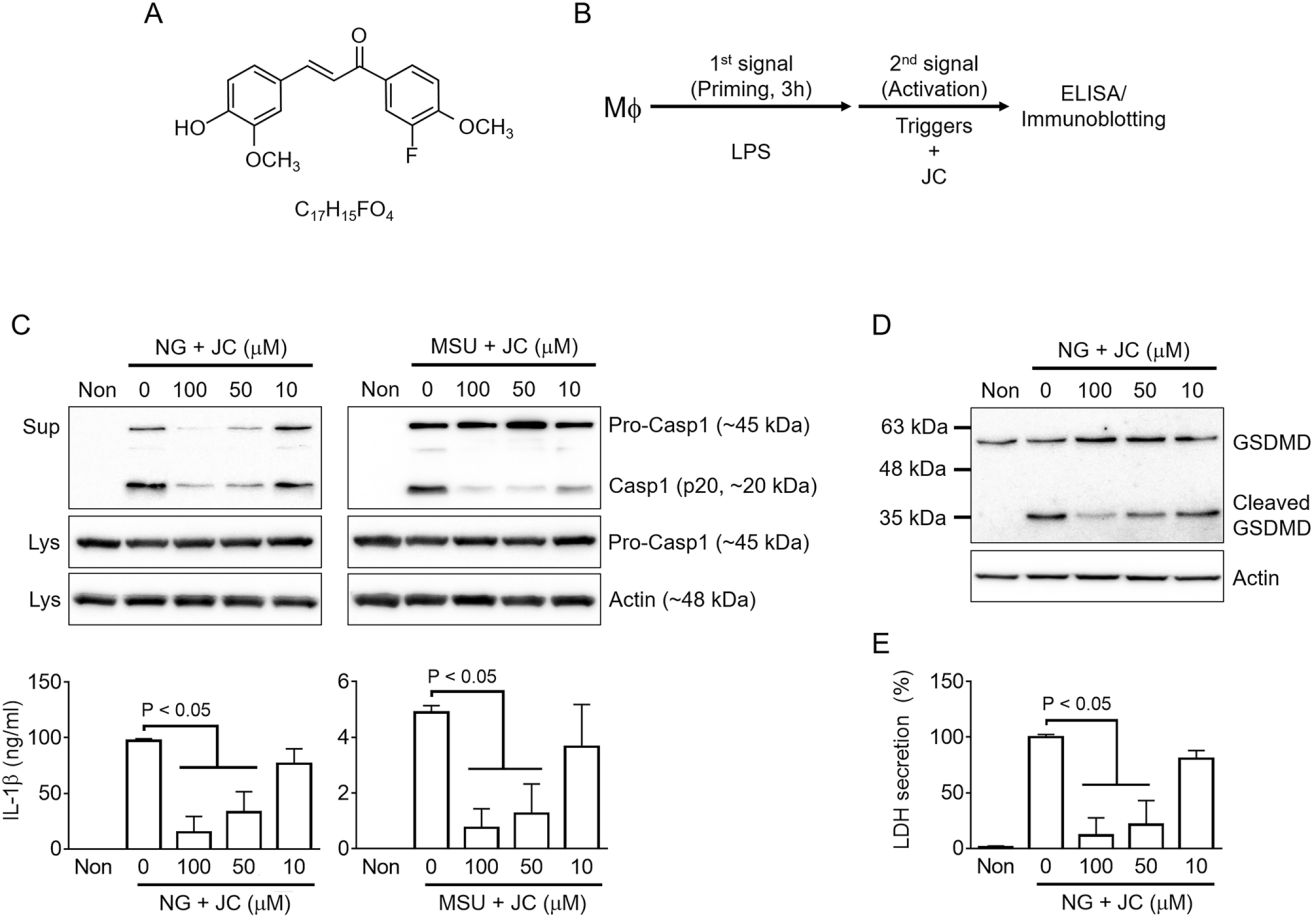
Effects of JC on NLRP3 inflammasome activation. (**A**) Chemical structure of JC2-11 (JC), a chalcone derivative. (**B**) Schematic diagram of the experimental mode of inflammasome activation. Macrophages (i.e., BMDM and PMA-treated THP-1) were treated with LPS for the priming step, and the inflammasome triggers were then treated with JC as the indicated concentration. The inflammasome indices were analyzed by ELISA and immunoblotting. **C**, LPS-primed BMDMs were treated with the NLRP3 inflammasome triggers (i.e., NG and MSU) with JC, as indicated. The cleavage of caspase-1 (Casp1) was observed by immunoblotting, and the secretion of IL-1β was measured by ELISA. LPS-primed BMDMs were treated with NG and JC. GSDMD cleavage in the lysate (**D**) and LDH secretion (**E**) were analyzed by immunoblotting and a biochemical assay. The bar graph presents the mean ± SD with at least three independent experiments.

JC2-11 (JC, Fig. 1A), a benzylideneacetophenone derivative, is a synthetic compound derived from the chalcones (1,3-diarylpropenones)^21^. Chalcones, natural phenolic products, belong to the flavonoid family, including curcumin, yakuchinone, green-tea-derived polyphenols, and flavonoids^22^. These natural phenolic products are precursors of various flavonoids with biological and pharmacological properties, such as antioxidant, antitumor, and anti-inflammatory activity^22^. JC has been designed to increase the antioxidant and anti-inflammatory properties, and its potency has been evaluated^21,23,24^. Based on the anti-inflammatory and radical scavenging activity of JC, this study examined the effects of JC on inflammasome activation. Murine and human macrophages were stimulated by inflammasome triggers in the presence of JC, and the change of indices (e.g., cytokine secretion and caspase-1 maturation) of the inflammasome activation were observed. In addition, the inflammasome-regulating effect of JC using an animal model was verified in vivo. Furthermore, the effects of JC on the intracellular signaling pathway of inflammasome activation were examined. Overall, JC is a candidate anti-inflammasome compound.

## Results

### JC inhibits NLRP3 inflammasome activation

The effects of JC on NLRP3 inflammasome activation were examined by priming murine bone marrow-derived macrophages (BMDMs) with LPS for 3 h, and treating them with the NLRP3 inflammasome triggers (e.g., nigericin [NG] and monosodium urate crystals [MSU]) and JC (Fig. 1B). The secretion of caspase-1 and IL-1β, and the formation of ASC speck from the BMDMs were measured in the cellular supernatant as indices of the inflammasome activation. As shown in Fig. 1C, JC attenuated the secretion of the cleaved caspase-1 (p20) and the release of IL-1β from the NG or MSU-treated cells. Furthermore, JC blocked the ASC speck formation from the assembly of NLRP3 inflammasome (Supplemental Fig. 1A). Pyroptosis, the other indicator of the inflammasome activation, induces the release of cytosolic proteins (e.g., LDH) through the pores consisting of the cleaved GSDMD^4^. As shown in Fig. 1D,E, JC also attenuated GSDMD cleavage and LDH release in response to NG-inducing NLRP3 inflammasome activation. On the other hand, this study tested whether JC works alone to trigger NLRP3 inflammasome activation in LPS-primed BMDMs (Supplemental Fig. 1B). JC alone did not lead to the secretion of caspase-1 (p20) and IL-1β, suggesting that JC is not a trigger of the inflammasome activation. Furthermore, this study checked whether the JC treatment did not induce cytotoxicity in macrophages (Supplemental Fig. 1C). Based on these data, JC may be an inhibitor of NLRP3 inflammasome activation.

### JC attenuated NLRC4 and AIM2 inflammasome activation

**Figure 2:**
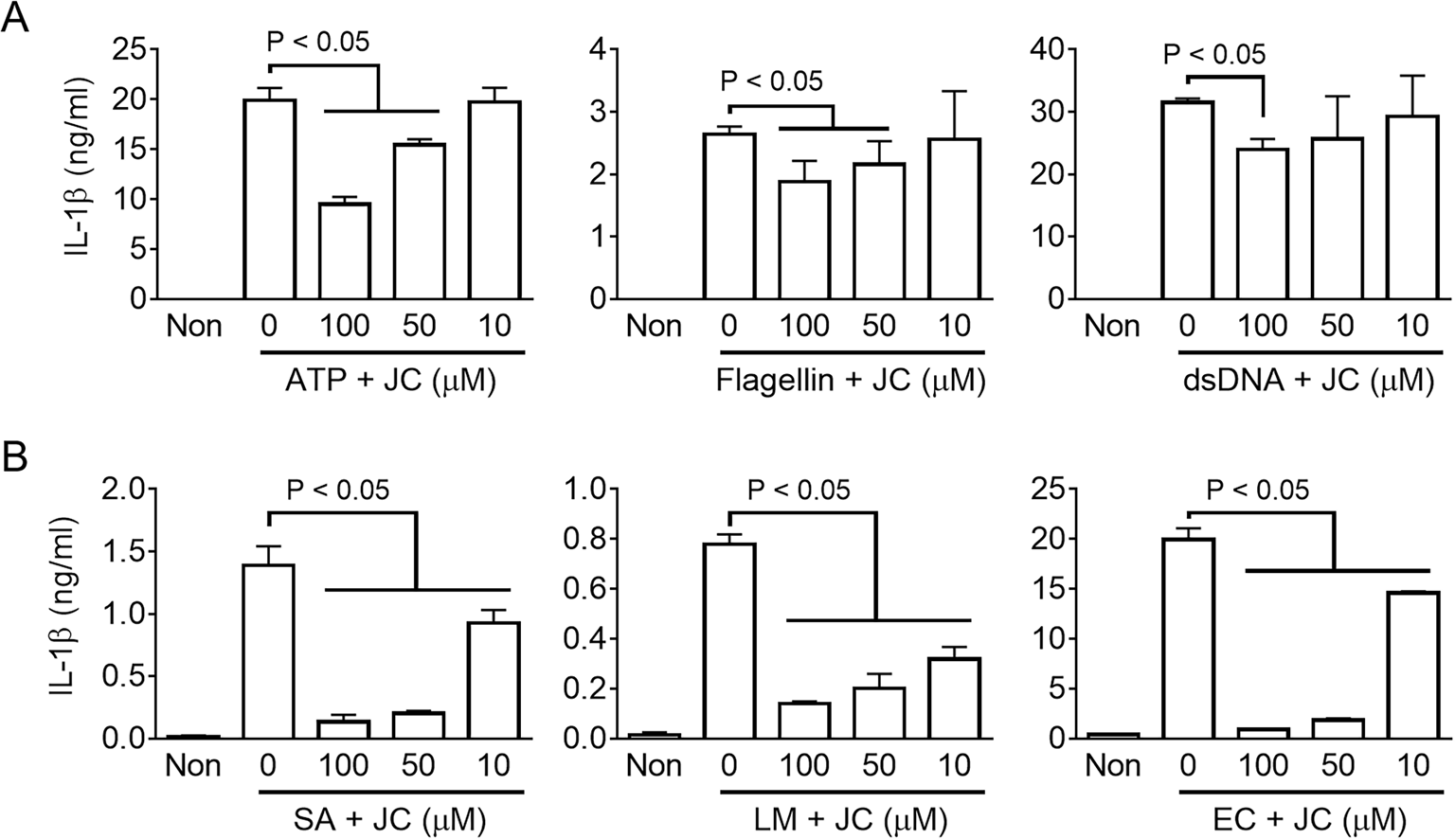
Effects of JC on NLRC4 and AIM2 inflammasomes. (**A**) LPS-primed BMDMs were treated with ATP, flagellin, and dsDNA to activate NLRP3, NLRC4, and AIM2 inflammasomes in the presence of JC, as indicated. (**B**) LPS-primed BMDMs were treated with JC, and inoculated with *Staphylococcus aureus* (SA), *Listeria monocytogenes* (LM), and *Escherichia coli* (EC). The secretion of IL-1β was measured by ELISA. The bar graph presents the mean ± SD with at least three independent experiments.

Further experiments were performed to determine if JC regulates other inflammasomes, such as NLRC4 and AIM2 inflammasomes. The LPS-primed BMDMs were activated the NLRP3, NLRC4, and AIM2 inflammasomes by ATP, flagellin, and dsDNA in the presence of JC. As shown in Fig. 2A and Supplemental Fig. 1D, JC attenuated IL-1β and LDH secretion elicited by ATP, flagellin, and dsDNA, suggesting that JC works as a pan-inhibitor of the NLRP3, NLRC4, and AIM2 inflammasomes. In addition, this study evaluated the effects of JC on inflammasome activation in macrophages infected with inflammasome triggering bacteria^25^. JC inhibited IL-1β secretion in response to *Staphylococcus aureus* (SA), *Listeria monocytogenes* (LM), and *Escherichia coli* (EC) (Fig. 2B). Overall, JC was identified as an inhibitor of NLRP3, NLRC4, and AIM2 inflammasome.

### Anti-inflammasome property of JC was confirmed in mice

**Figure 3:**
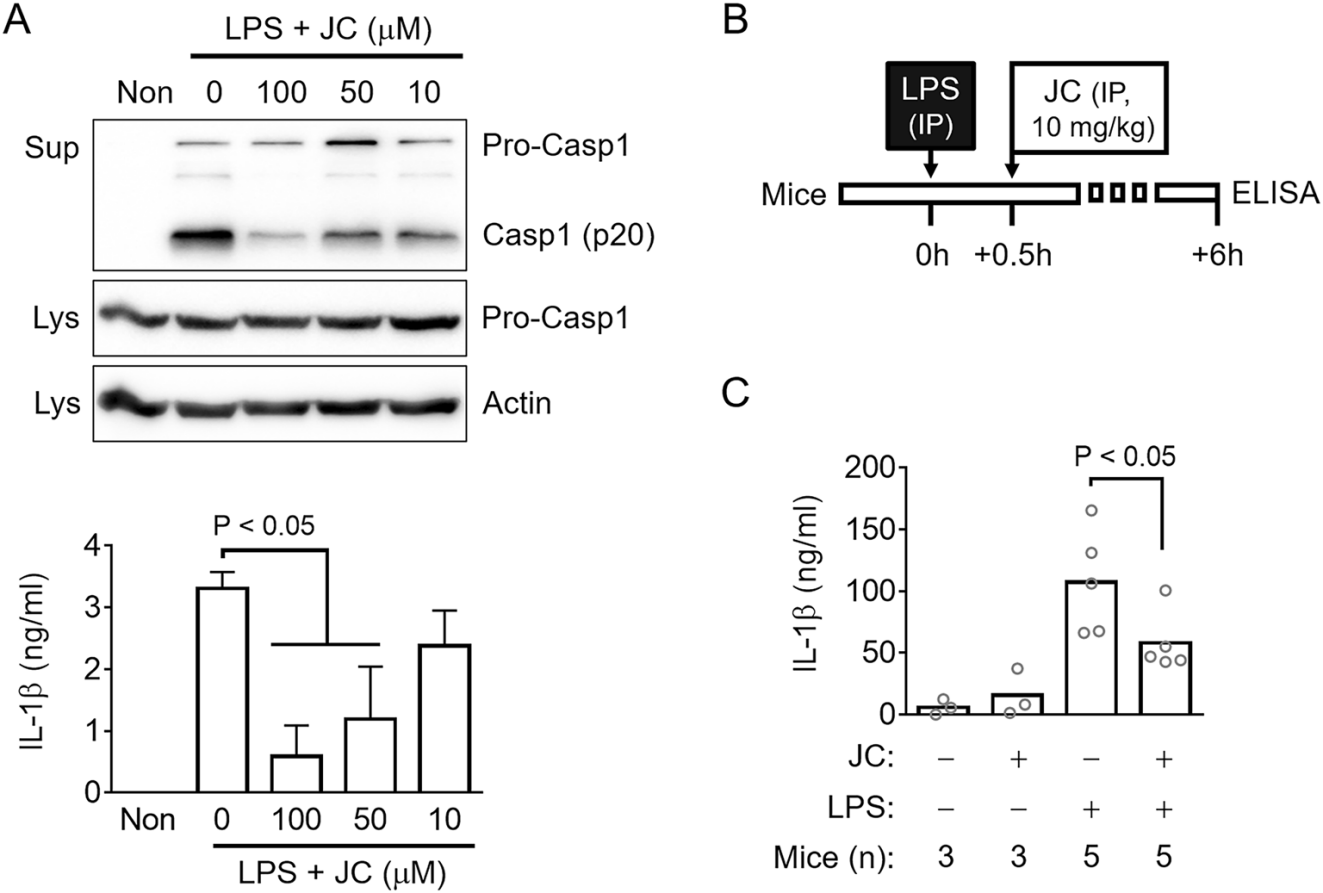
Effects of JC on NC inflammasome and LPS injected mice. (**A**) LPS-primed BMDMs were transfected with LPS and treated with JC. The cleavage of caspase-1 was analyzed by immunoblotting, and the release of IL-1β was detected by ELISA. The bar graph presents the mean ± SD with at least three independent experiments. (**B**) Schematic diagram of the experimental process. The mice were injected intraperitoneally (IP) with LPS and JC at the indicated times. A peritoneal lavage was collected to measure IL-1β secretion 6 h after the LPS injection. (**C**) The peritoneal IL-1β secretion was measured by ELISA. The dot indicated the value of each mouse.

The introduction of LPS into the cytoplasm of macrophages activates a non-canonical (NC) inflammasome, and an injection of LPS into the peritoneal cavity of mice is an animal model for NC inflammasome^7,26^. Thus, the inhibitory effects of JC on the NC inflammasome activation were examined in macrophages and mice. First, the LPS-primed BMDMs were transfected with LPS to activate NC inflammasome in the presence of JC. JC significantly inhibited the release of caspase-1 (p20) and IL-1β from the LPS transfected cells (Fig. 3A). Furthermore, JC also attenuated GSDMD cleavage (Supplemental Fig. 2A). A sepsis model injected with LPS was established, and JC was administrated to the sepsis mice to confirm the anti-inflammatory properties of JC (Fig. 3B). The LPS injection increased peritoneal IL-1β secretion, which was attenuated by JC administration (Fig. 3C). On the other hand, no improvement of survival rate was observed in the mice injected with JC plus a lethal dosage of LPS when compared with the mice injected only a lethal dosage of LPS (Supplemental Fig. 2B). Overall, JC inhibited IL-1β secretion in cells and mice.

### JC inhibits the inflammasome activation in human macrophages

**Figure 4:**
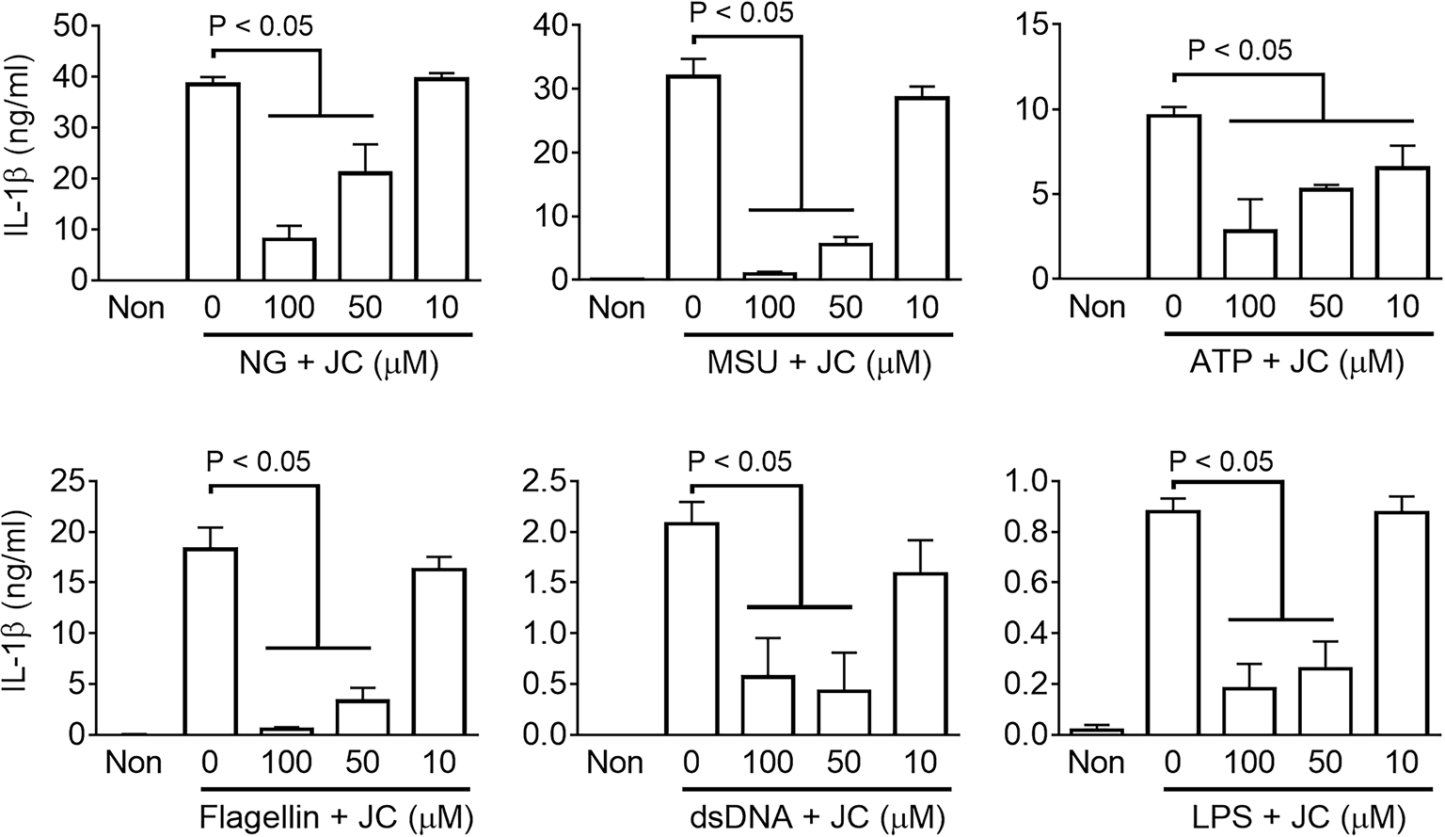
Effects of JC on the human inflammasome activation. PMA-treated THP-1 cells, a human macrophage, were primed with LPS, and NLRP3, NLRC4, AIM2, and NC inflammasome were then activated by a treatment with NG, MSU, and ATP, as well as the transfection of flagellin, dsDNA, and LPS with/without JC. The secretion of IL-1β was measured by ELISA. The bar graph presents the mean ± SD with at least three independent experiments.

The inhibitory effect of JC on the inflammasome activation in murine cells was revealed. Therefore, human macrophages were used to test the anti-inflammasome property of JC. A human monocyte-like cell line (THP-1) was treated with PMA to differentiate into macrophage-like cells and subjected to the inflammasome activation process (Fig. 1B). The PMA-treated THP-1 cells were primed with LPS, and the NLRP3 (ATP, MSU, and ATP), AIM2 (dsDNA), NLRC4 (flagellin), and NC (LPS) inflammasomes were then activated using the selective triggers. Simultaneously, the cells were co-treated with JC to assess its anti-inflammasome activity, and IL-1β secretion was measured as the readout of the inflammasome activation. JC inhibited all inflammasome activation in human macrophages (Fig. 4). Based on these results, JC was disclosed as an inflammasome inhibitor.

### JC inhibits the inflammasome activation by disrupting mitochondrial ROS production and caspase-1 activity

**Figure 5:**
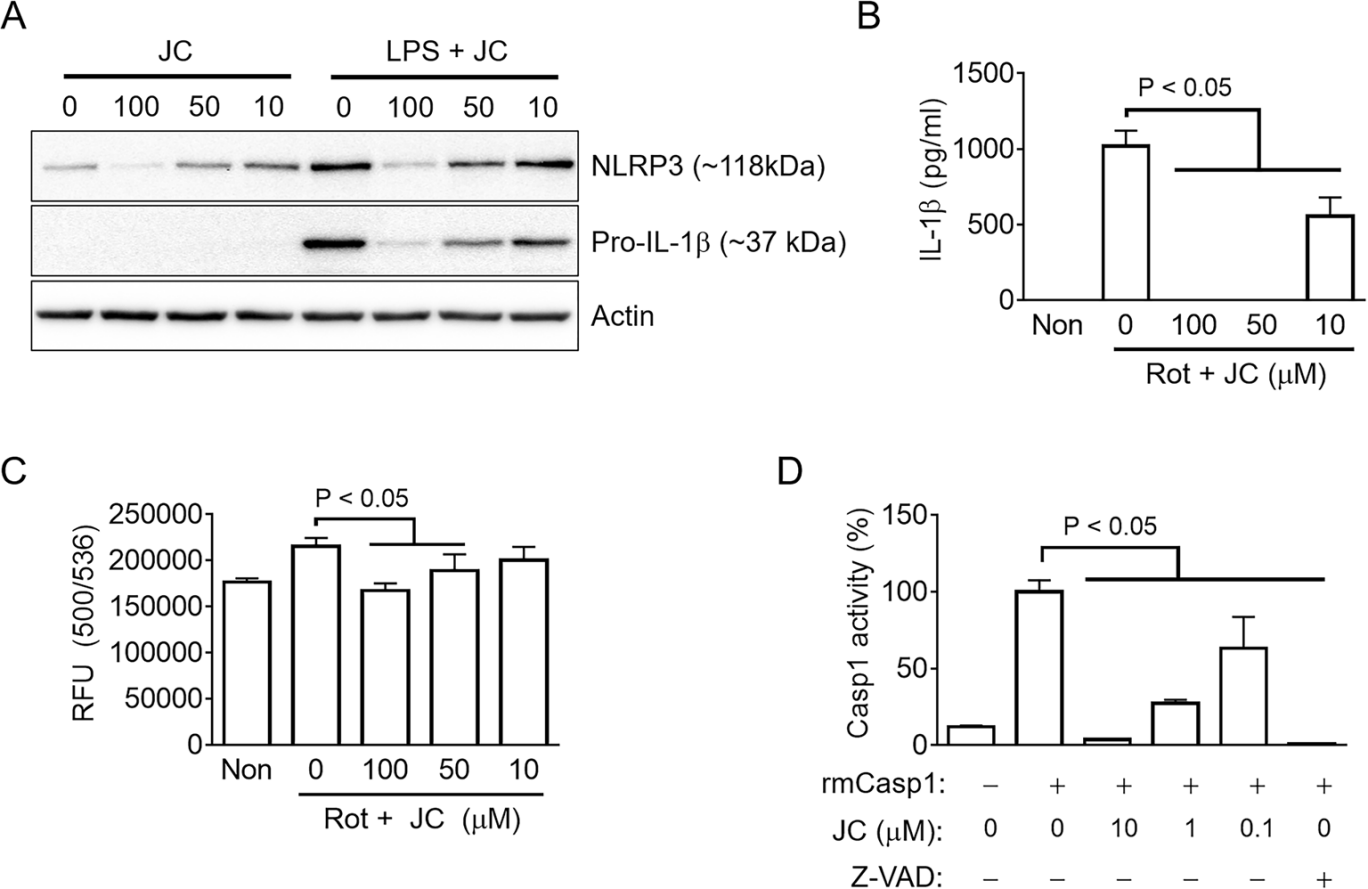
Mechanistic study of the anti-inflammasome property of JC. (**A**) BMDMs were treated with LPS (10 ng/ml) and JC for 3 h, and the protein levels of NLRP3 and pro-IL-1β were analyzed by immunoblotting. (**B**) LPS-primed BMDMs were treated with JC and rotenone (Rot), which led to mitochondrial ROS production. The IL-1β released was measured by ELISA. (**C**) LPS-primed BMDMs were treated with Rot and JC, and the ROS levels were then analyzed using a relative fluorescence unit (RFU, excitation at 505 nm, and emission at 536 nm). (**D**) The activity of recombinant mouse caspase-1 (rmCasp1) was measured using an assay kit in the presence of JC and Z-VAD-FMK (Z-VAD, pan-caspase inhibitor). The bar graph presents the mean ± SD with at least three independent experiments.

This study examined how JC inhibits inflammasome activation and which intracellular inflammasome signaling is disrupted by JC. For successful inflammasome activation, the up-regulation of the inflammasome components and substrates needs to occur before the inflammasome assembly^27^. Thus, this study tested the effect of JC on NLRP3 and pro-IL-1β expression during the priming step (Fig. 5A). As a result, JC interrupted NLRP3 and pro-IL-1β expression in the BMDM treated with LPS, leading to the 1^st^ signal of inflammasome activation. Furthermore, JC also inhibited the transcription of pro-inflammatory cytokines in the LPS-treated macrophages (Supplemental Fig. 3A). The production of mitochondrial ROS is a well-known intracellular signal to induce NLRP3 inflammasome assembly^5,28^. Thus, this study tested the role of JC on the LPS-primed BMDMs treated with rotenone (Rot) to elicit mitochondrial ROS production^5,28^. JC blocked the secretion of IL-1β and caspase-1 (p20) induced by Rot (Fig. 5B and Supplemental Fig. 3B). JC also attenuated the increase in ROS by Rot (Fig. 5C). The effects of JC on the activity of caspase-1, the effector protein of the inflammasome activation, were examined further. JC significantly inhibited the activity of recombinant caspase-1 in mice (Fig. 5D and Supplemental Fig. 3C) and humans (Supplemental Fig. 3D), whereas JC did not change the activity of caspase-4 (Supplemental Fig. 3E). Overall, JC regulates the inflammasome activation by inhibiting several intracellular signals, such as the priming step, mitochondrial ROS production, and caspase-1 activity.

## Discussion

In this study, JC, a chalcone analog, has an anti-inflammasome effect in human and murine macrophages. JC significantly inhibited several inflammasome readouts (e.g., the secretion of IL-1β, caspase-1, and LDH, and the cleavage of caspase-1, and GSDMD) in response to selective NLRP3, NLRC4, AIM2, and NC inflammasome triggers. In addition, JC attenuated the IL-1β secretion from macrophages infected by inflammasome triggering bacteria. These inhibitory properties of JC were confirmed using an in vivo model; JC inhibited the peritoneal IL-1β releases from the LPS-injected mice. Although the effects of flavonoids and chalcones on inflammasome have only been studied using NLRP3 inflammasome, it was suggested that JC as a chalcone works as an inhibitor of NLRC4, AIM2, and NC inflammasome through the direct inhibition of caspase-1. In the current study, JC inhibited both the inflammasome activation and mitochondrial ROS production caused by rotenone-mediated mitochondrial dysfunction. In addition, JC disrupted the priming step; JC interrupted the gene expression of inflammasome components and pro-inflammatory cytokines. Overall, JC, a benzylideneacetophenone derivative, is a candidate for inflammasome regulators.

Natural molecules, including chalcones and flavonoids found in fruit and vegetables, are well-known free radical scavengers, which have a beneficial effect on the pathological aspect. Chalcone derivatives were synthesized, and their biological properties were tested to enhance this advantage^21–24,29,30^. The current tested molecule, JC, was one of the derivatives synthesized, and its antioxidant effect was ranked the top among the derivatives^21^. The radical scavenging activity of JC was two-time stronger than that of resveratrol, and it significantly inhibited nitric oxide generation in response to LPS^21^. JC was also suggested to be a neuroprotective compound because it reduced ROS production in neuroblastoma cell lines^24^. Similar derivatives to JC have anti-cancer effects in prostate cancer cells, and its neuroprotective activity was studied in the animal model of Parkinson’s diseases^22,29^. These biological effects of JC and its analogs are due to their antioxidant activity^21,22,29^. Thus, JC could inhibit the activation of NLRP3 inflammasome because one of the NLRP3 triggers is ROS^28^. Interestingly, JC has an inhibitory role on caspase-1, suggesting that JC may be a pan-inhibitor of inflammasome activation.

Chalcones have various pharmacological effects on immunity, inflammation, tumor, and infection^31^. The effect of chalcones on the inflammasome activation was also investigated as follows. Isoliquiritigenin, a chalcone from *Glycyrrhiza uralensis*, is an anti-NLRP3 agent that blocked the secretion of IL-1β and the formation of ASC oligomerization induced by NLRP3 triggers^32,33^. Isoliquiritigenin alleviates the metabolic syndrome induced by high-fat diets in mice by inhibiting NLRP3 inflammasome signaling and the symptom induced by intracerebral hemorrhage^32,33^. Cardamonin, a natural chalcone, was assessed as an inhibitor of NLRP3 inflammasome signaling, including up-regulation of the *Nlrp3* and *IL-1β* genes in cardiac valve cells and chondrocytes^34,35^. 1,3-Diphenyl-2-propen-1-one, a trans-chalcone, down-regulates the gene expression of pro-inflammatory and inflammasome components by inhibiting the NF-κB signal pathway. In addition, it attenuated the IL-1β secretion from the macrophages stimulated by MSU^19^. This trans-chalcone also ameliorated the symptoms of the MSU-mediated acute gout model, a mouse model of NLRP3 inflammasome-mediated disease^19^. Overall, the inhibitory effect of chalcone on inflammasomes has been assessed mainly using NLRP3, and the inhibitory mechanism is unclear.

The NLRP3 inflammasome has been regarded as a critical signal for the pathogenesis of metabolic and degenerative diseases, such as type 2 diabetes, arteriosclerosis, and Alzheimer’s diseases^3^. Thus, research has been actively conducted to identify a potent inhibitor of NLRP3 inflammasome from natural compounds, such as chalcones. Using computational modeling, synthetic chalcones were designed, and their anti-inflammasome properties were evaluated^36^. The selected chemicals, tertiary sulfonylurea compounds, inhibited NLRP3 and AIM2 inflammasome activation^36^. In addition, the chalcone analog screened by the hit-to-lead attenuated the symptoms in the NLRP3 inflammasome-mediating diseases models, such as dextran sulfate sodium-induced colitis and LPS-induced sepsis^20^. Several chalcone analogs were synthesized and screened by the structure–activity relationship for the potent NLRP3 inhibitor^16^. 11Cha1, a selected analog, induced the proteolytic degradation of IκBα and the nuclear translocation of NF-κB. In addition, it attenuated NLRP3 inflammasome by inhibiting K^+^ efflux^16^. In addition, chalcone analogs were screened to pick up an NLRP3 inhibitor using *Helicobacter pylori*-infected macrophages^15^. The candidate compound inhibited IL-1β, IL-18, and caspase-1 secretion by interrupting the interleukin-1 receptor-associated kinase 4 (IRAK4) and the NF-κB signaling pathways^15^. Flavonoids, including a chalcone isolated from *Millettia velutina*, were tested for their anti-NLRP3 properties^18^. Velutone F, a chalcone, inhibited NLRP3 inflammasome activation without altering the priming step, and it enhanced the survival rate of LPS septic shock in mice^18^. The screening study was also conducted on flavonoids. Among 56 flavonoids, apigenin was selected; it attenuated the activation of NLRP3 and AIM2 inflammasomes but not NLRC4 inflammasome^17^. Apigenin disrupted the phosphorylation of Syk and Pky2, and attenuated MSU-induced peritonitis^17^. Based on these studies, several chalcones were characterized as an inhibitor of NLRP3 and AIM2 inflammasomes, and their effect on the priming step was varied. The chalcone studied in this paper, JC, exhibited unique properties on inflammasome activation. JC inhibited the priming step and attenuated NLRP3, NLRC4, AIM2, and NC inflammasome activation by inhibiting ROS production and caspase-1 activity.

## Materials and methods

### Cell culturing

Unless indicated otherwise, all cell culture products and plastics were supplied by WELGENE (Gyeongsanbuk-do, Republic of Korea) and SPL LIFE SCIENCE (Gyeonggi-do, Republic of Korea). For BMDMs, bone marrow cells were isolated from the tibia and femur of mice (C57BL/6 mice, 6–12 weeks old, NARA BIOTECH, Seoul, Republic of Korea), and incubated in the DMEM media containing fetal bovine serum (FBS, 10%), antibiotics and L929 cell-conditioned media (30%, a source of a macrophage colony-stimulating factor) for 7 days. Human monocyte-like cells (THP-1; KOREA CELL LINE BANK, Seoul, Republic of Korea) were differentiated into macrophages in RPMI 1640 (RPMI) containing FBS (10%), antibiotics, and phorbol 12-myristate 13-acetate (200 nM, PMA; INVIVOGEN, San Diego, CA, USA) for 24 h. All cells were incubated at 37 °C in an atmosphere containing 5% CO_2_.

### Cell treatment

The BMDM and PMA-treated THP-1 cells (1 × 10^6^ cells/well in 12-well-plate) were primed with LPS (1 μg/mL, L4130, SIGMA-ALDRICH Co., St. Louis, MO, USA) for 3 h, and the media were replaced with RPMI including an inflammasome trigger with/without JC (Fig. 1A). The synthetic process of JC was fully described and identified by Fourier transform infrared (FTIR) and nuclear magnetic resonance (NMR) spectroscopy, including high-resolution mass spectroscopy^21^. JC was dissolved in the dimethyl sulfoxide (DMSO) and diluted with media as described^24^. The treatment modes of the inflammasome trigger were described as follows^6,37^: nigericin (NG, 40 μM, TOCRIS BIOSCIENCE, Bristol, UK) for 1 h; monosodium urate crystals (MSU, 400 μg/mL, SIGMA-ALDRICH Co.) for 3 h; adenosine triphosphate (ATP, 5 mM, Invivogen) for 1 h; flagellin (500 ng/mL; INVIVOGEN) with Lipofectamine 2000 (10 μL/mL, INVITROGEN, Carlsbad, CA, USA) for 3 h; dsDNA (1 μg/mL) with jetPRIME (2 μL/mL, POLYPLUS-TRANSFECTION Inc., Illkirch, France) for 1 h; LPS (2 μg/mL, SIGMA-ALDRICH Co.) with FuGENE HD (2.5 μL/mL, ROCHE, Penzberg, Germany) for 6 h; rotenone (160 μM, SANTA CRUZ BIOTECHNOLOGY, Dallas, TX, USA) for 6 h; *Staphylococcus aureus* (SA, multiplicity of infection [MOI] 35) for 6 h; *Listeria monocytogenes* (LM, MOI 35) for 3 h; *Escherichia coli* (EC, MOI 10) for 6 h. The bacteria were cultured at 37 °C on Luria–Bertani media (CONDALAB, Torrejón de Ardoz, Madrid, Spain) for EC, or Brain Heart Infusion media (CONDALAB) for SA and LM^6^. The growth of bacteria was assessed by measuring the optical density at 600 nm using a spectrophotometer.

After inflammasome activation, the supernatants (Sup) were collected for further assays. The remaining cells were lysed with a lysis buffer containing Triton X-100 (0.01%), NaCl (150 mM), Tris-base (50 mM, pH 8.0), and proteinase inhibitors (Halt cocktail, THERMOFISHER SCIENTIFIC), and the lysates (Lys) were then collected after centrifugation^6^.

### Western blotting

The Sup and Lys were separated by electrophoresis using 10 or 16% SDS-PAGE and transferred to a membrane (PVDF: GE HEALTHCARE BIO-SCIENCE, Pittsburgh, PA, USA)^6^. The membranes were proved overnight at 4 °C as follows: anti- caspase-1 (p20) antibody (AG-20B-0042-C100, ADIPOGEN Co., San Diego, CA, USA), anti-GSDMD antibody (ab209845, ABCAM, Cambridge, MA, USA), anti-NLRP3 antibody (AG-20B-0014-C100, ADIPOGEN Co.), anti-mouse IL-1β antibody (AF-401-NA, R&D SYSTEMS), or anti-Actin antibody (sc-1615, SANTA CRUZ BIOTECHNOLOGY). The membrane was incubated with the secondary antibodies conjugated with horseradish peroxidase (INVITROGEN for anti-mouse antibodies, ABCAM for anti-gout antibodies) for 2 h at room temperature. Immunoblotting images were obtained using a chemiluminescent system (EZ-Capture II, ATTO TECHNOLOGY) and a chemiluminescence solution (WESTSAVER STAR, ABFRONTIER, Seoul, Republic of Korea). Supplementary information presents the full-length bolts for figures.

### Animal study

Female mice (C57BL/6, 8-week-old, NARA BIOTECH) were divided into four groups, and injected intraperitoneally with LPS (100 μg/mouse) or JC (250 μg/mouse), or both, as shown in Fig. 3B. Six hours after the initial injection, the mice were sacrificed by CO_2_ inhalation. Their peritoneal cavities were flushed with phosphate buffered saline (5 mL), and collected the peritoneal lavages for further analysis. The mice were supplied with normal chow and water ad libitum, and raised at room temperature (18–24 °C) and with a 12 h light/dark cycle. All animal experiments were carried out in accordance with the National Institutes of Health Guide for the Care and Use of Laboratory Animals, and the Animal research: Reporting of In Vivo Experiments (ARRIVE) guidelines. The experimental protocols were approved by the Institutional Animal Care and Use Committee of Kangwon National University (IACUC; approval no. KW-210317-2 and KW-220401-4).

### Assay for IL-1β secretion, LDH release, and caspase-1 activity

The IL-1β levels in the cellular supernatants and peritoneal lavages were measured using an enzyme-linked immunosorbent assay (ELISA) kit (DY201 or DY401, R&D SYSTEMS). LDH secretion was analyzed using an assay kit (BCT-LDHP, BIOMAX, Seoul, Republic of Korea). For the ROS measurement, LPS-primed BMDMs (1.25 × 10^5^ cells/well in a 96-well-black plate) were treated with dihydrorhodamine 123 (DHR123, CAYMAN CHEMICAL, Ann Arbor, MI, USA) in the presence of rotenone (160 μM) and JC for 6 h. The caspase-1 activity was determined using mouse recombinant caspase-1 (one unit per reaction, BIOVISION, Milpitas, CA, USA) and a caspase-1 fluorometric assay kit (BIOVISION) in the presence of JC or the pan-caspase inhibitor (Z-VAD-FMK, 10 μg/mL; R&D SYSTEMS). The recombinant caspase-1 was co-incubated JC or Z-VAD-FMK in the reaction buffer containing YVAD-AFC for 1 h at 37 °C according to the manufacturer's protocol. The plates were analyzed using a multi-microplate spectrophotometer (Synergy H1 Hybrid Multi-Mode Reader, BIOTEK, Winooski, VT, USA).

### Data analyses

Statistical analyses were conducted using software (GRAPHPAD PRISM 6, San Diego, CA) as follows: Mann–Whitney test or Kruskal–Wallis test. The p-value is shown in the figures.

## Supplementary Information

Supplementary Figures.

## Data Availability

The datasets used and/or analysed during the current study available from the corresponding author on reasonable request.
