# Supplementary material for: JC2-11, a benzylideneacetophenone derivative, attenuates inflammasome activation
Source: Sci Rep. 2022 Dec 28;12:22484. doi: 10.1038/s41598-022-27129-3 (PMC9797494; doi:10.1038/s41598-022-27129-3)

Supplementary Information

JC2-11, a benzylideneacetophenone derivative, attenuates inflammasome activation

Gilyoung Lee[1, #], Huijeong Ahn[1, #], Jang-Hyuk Yun[1], Jeongho Park[1], Eunsong Lee[1], Seikwan Oh[2] and Geun-Shik Lee[1,*]

[1]College of Veterinary Medicine and Institute of Veterinary Science, Kangwon National University, Chuncheon, Gangwon, 24341, Republic of Korea.

[2]Department of Neuroscience and Medical Research Institute, School of Medicine, Ewha Womans University, Seoul 03760, Republic of Korea.

*Correspondence: Geun-Shik Lee, D. V. M., Ph. D.

Laboratory of Inflammatory Diseases, Department of Physiology, College of Veterinary Medicine, Kangwon National University, Chuncheon, Gangwon, 24341, Republic of Korea.

e-mail: leegeun@kangwon.ac.kr, Tel: +82-33-250-8683, Fax: +82-33-244-2367

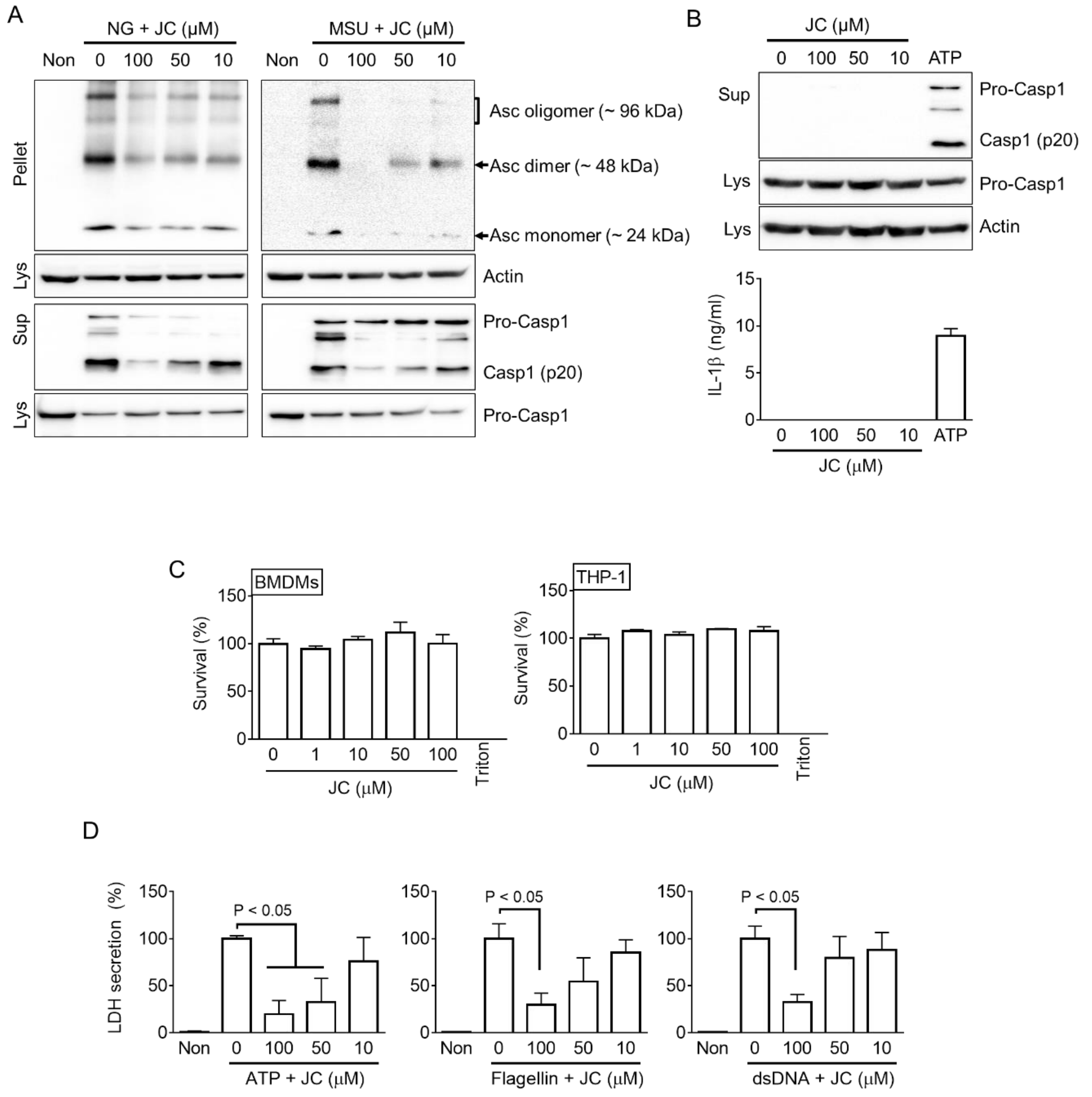

Supplemental Fig. 1.

A, LPS-primed BMDMs were treated with NG and MSU in the presence of JC, and the formation of ASC speck and the secretion of caspase-1 were analyzed by immunoblotting. To analyze ASC speck formation, the lysate (Lys) of BMDMs was transferred into a new tube and collected by centrifugation at 15,000 rcf for 5 min. The remaining pellet was washed twice with PBS and then re-suspended and cross-linked with 2 mM suberic acid bis (SIGMA-ALDRICH Co.) for 1 h, followed by centrifugation at 15,000 rcf for 5 min. The cross-linked

pellets (Pellet) were re-suspended in 50 μL of 2 × loading dye buffer (116 mM Tris, 3.4 % SDS, 12 % glycerol, 200 mM DTT, 0.003 % bromophenol blue) [Ref.1, 2]. The pellet was subjected to Western blot assay using anti-ASC antibody (sc-22514, SANTA CRUS BIOTECHNOLOGY, Dallas, TX, USA). **B,** LPS-primed BMDMs were treated with JC as indicated, and the Casp1 cleavage and the IL-1β secretion were measured by immunoblotting and ELISA. **C**, BMDMs and PMA-treated THP-1 cells were primed with LPS and then treated with JC, as indicated. The survival rates were monitored using an assay kit (EZ-Cytox, DOGEN BIO, Seoul, Republic of Korea) according to the manufacturer's method. The survival of non-treated groups (Non) was set to 100%, and the survival of triton X-100 (Triton, 0.01%) was put at 0%. **D**, LPS-primed BMDMs were treated with ATP, and transfected with flagellin and dsDNA. LDH secretion was analyzed using a biochemical assay. The bar graph presents the mean ± SD with at least three independent experiments.

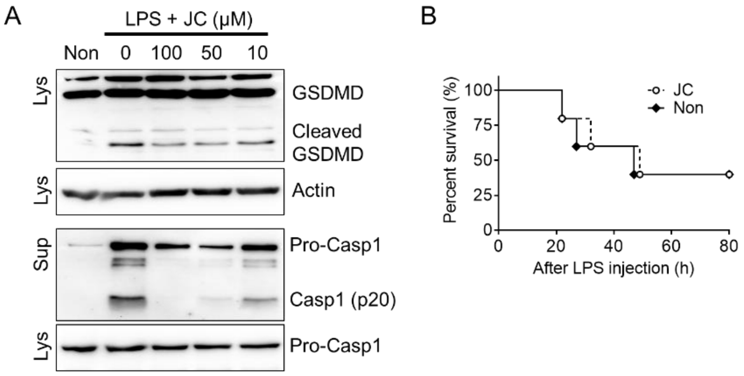

Supplemental Fig. 2.

A, LPS-primed BMDMs were transfected with LPS in the presence of JC. GSDMD cleavage in the lysate and caspase-1 (Casp1) secretion in the supernatant were analyzed by immunoblotting. **B**. Mice (n=10 per each group, total n=20; female, C57BL/6, eight weeks old, NARA BIOTECH) were injected intraperitoneally with LPS (500 μg/mouse), and 30 min later, JC (25 μg/mouse) was administered in the peritoneum. The survival was checked every eight hours.

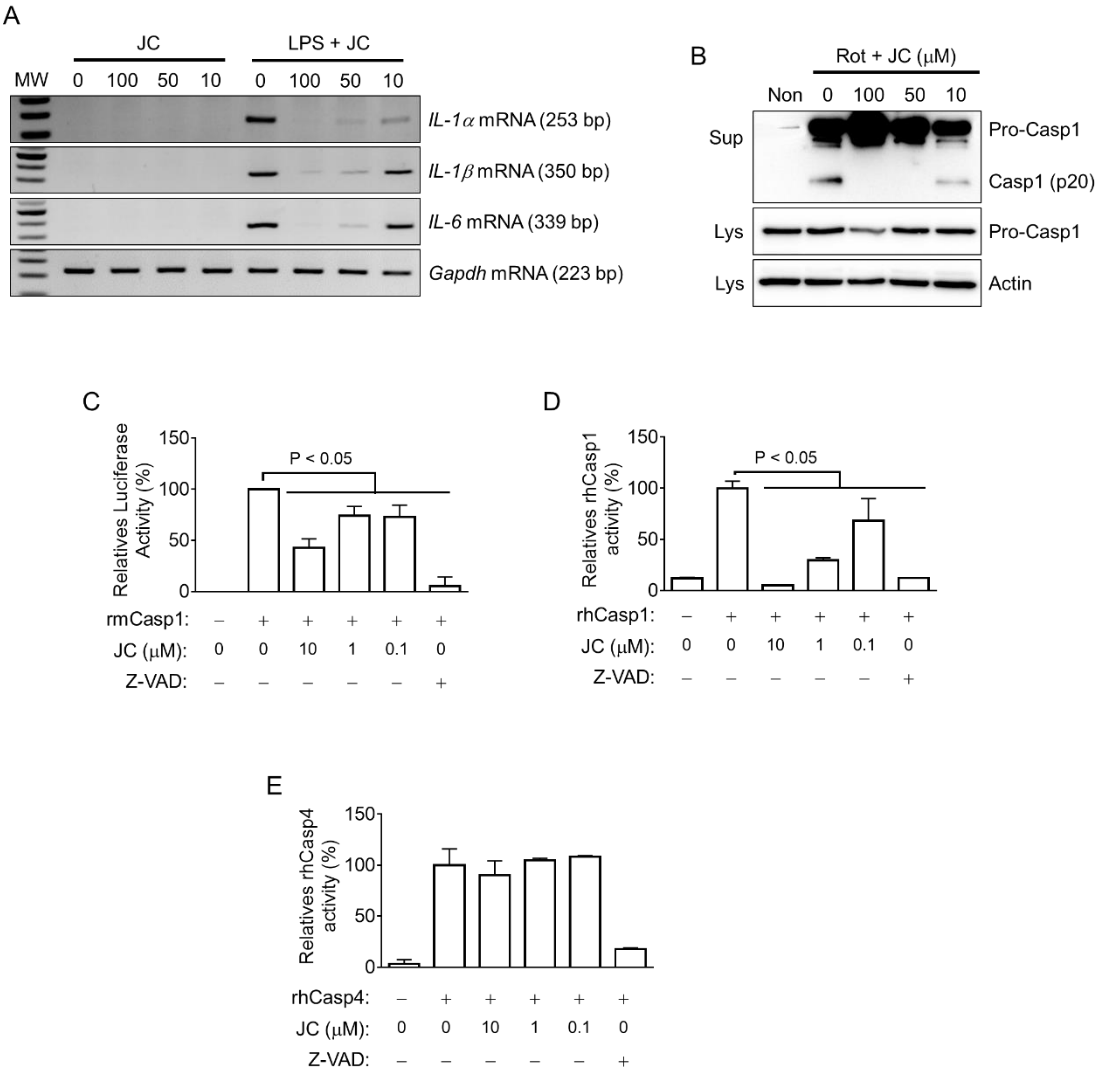

Supplemental Fig. 3.

A, BMDMs were treated with LPS (10 ng/ml) for 3h. The total RNAs were then extracted with a reagent (NucleoZOL, MACHEREY-NAGEL GmbH & Co. KG, Postfach, Düren, Germany) and synthesized into cDNA using M-MLV reverse transcriptase (ENZYNOMICS Co., Daejeon, Korea). The transcription was amplified using a SimpliAmp Thermal Cycler (THERMO FISHER SCIENTIFIC), nTaq polymerase (ENZYNOMICS), and specific gene primers as follows: *IL-1α* (Gene Bank ID, NM_010554), 5'-GAA GCT CGT CAG GCA GAA GT-3' and 5'-TGT TTC TGG CAA CTC CTT CA-3'; *pro-IL-1β* (NM_008361), 5'-CAG GCA GGC

AGT ATC ACT CA-3’ and 5’-AGG CCA CAG GTA TTT TGT CG-3’; *IL-6* (NM_031168), 5’-GTT CTC TGG GAA ATC GTG GA-3’ and 5’-GGA AAT TGG GGT AGG AAG GA-3’; *Gapdh* (NM_001289726, 223 bp), 5’-AAC TTT GGC ATT GTG GAA GG-3’ and 5’-ACA CAT TGG GGG TAG GAA CA-3’. The PCR products were visualized by agarose gel electrophoresis and ethidium bromide staining. **B**, LPS-primed BMDMs were treated with rotenone (Rot) and JC, as indicated. The Casp1 cleavages were observed by immunoblotting. **C**, The effects of JC on the activity of rmCasp1 were measured using a luciferase-based assay kit (Caspase-Glo 1 Inflammasome Assay, PROMEGA, Madison, WI, USA) according to the manufacturer’s protocol. **D** and **E**, The activities of recombinant human Casp1 (D; rhCasp1, BIOVISION) and recombinant human caspase-4 (E; rhCasp4, BIOVISION) were measured using a fluorescence-based assay kit (Caspase-1 Fluorometric Assay Kit, BIOVISION) and Ac-LEVD-AMC (caspase-4 substrate; ENZO LIFE SCIENCES Inc. Farmingdale, NY, USA) in the presence of JC and Z-VAD-FMK (Z-VAD, a pan-Casp1 inhibitor). The bar graph presents the mean ± SD with at least three independent experiments.

Full-length blots of Figure 1C

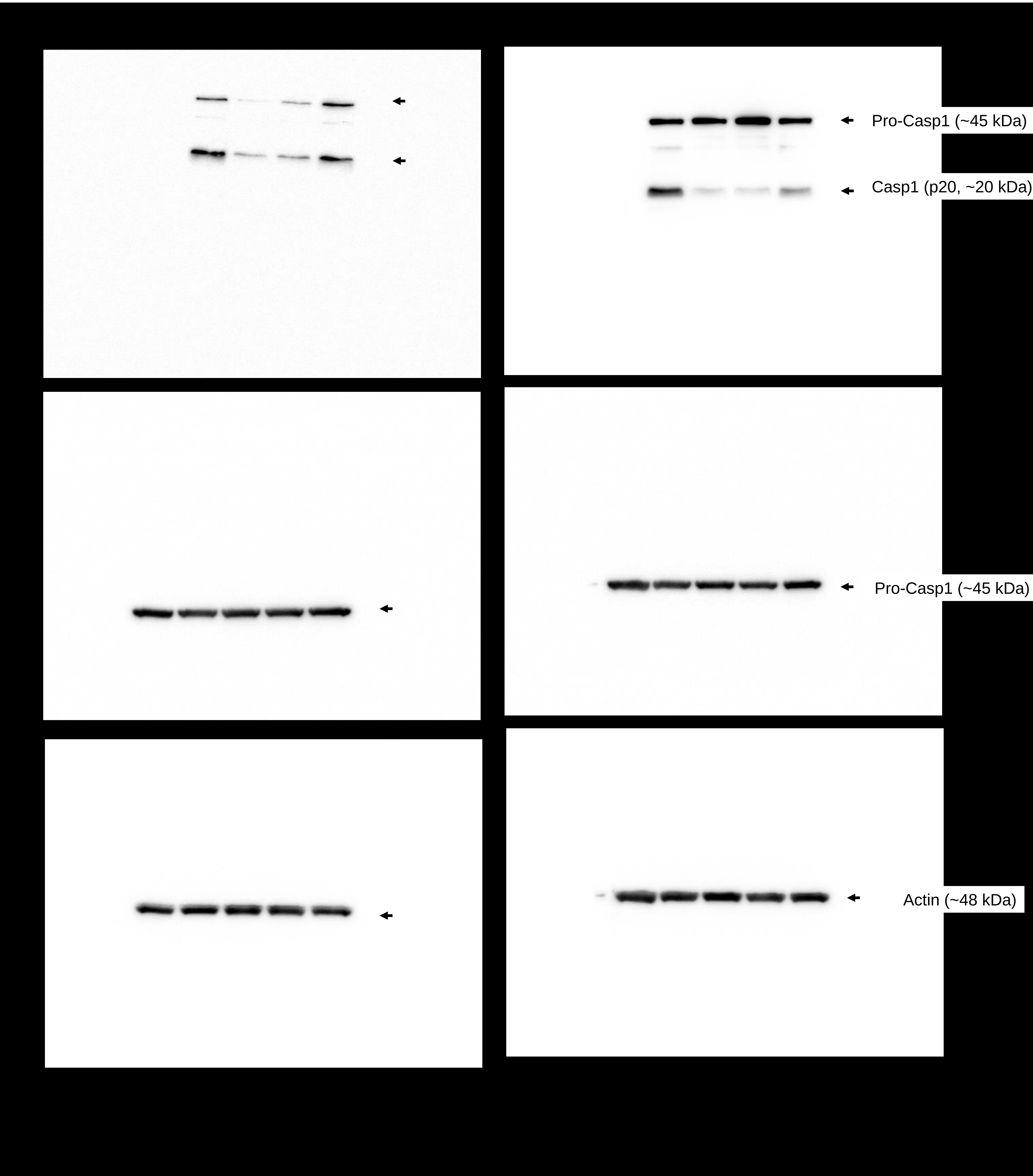

Full-length blots of Figure 1D

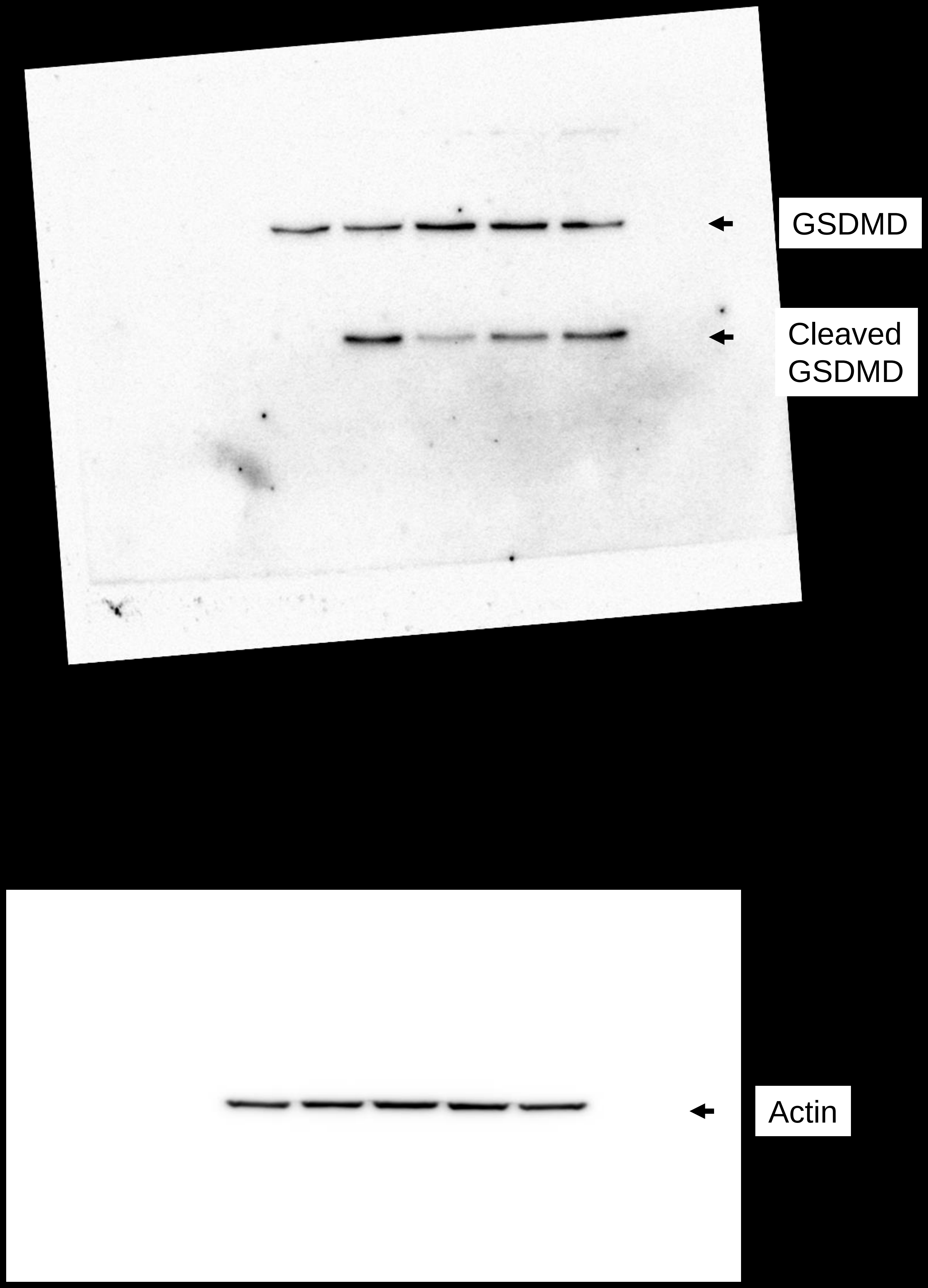

Full-length blots of Figure 3A

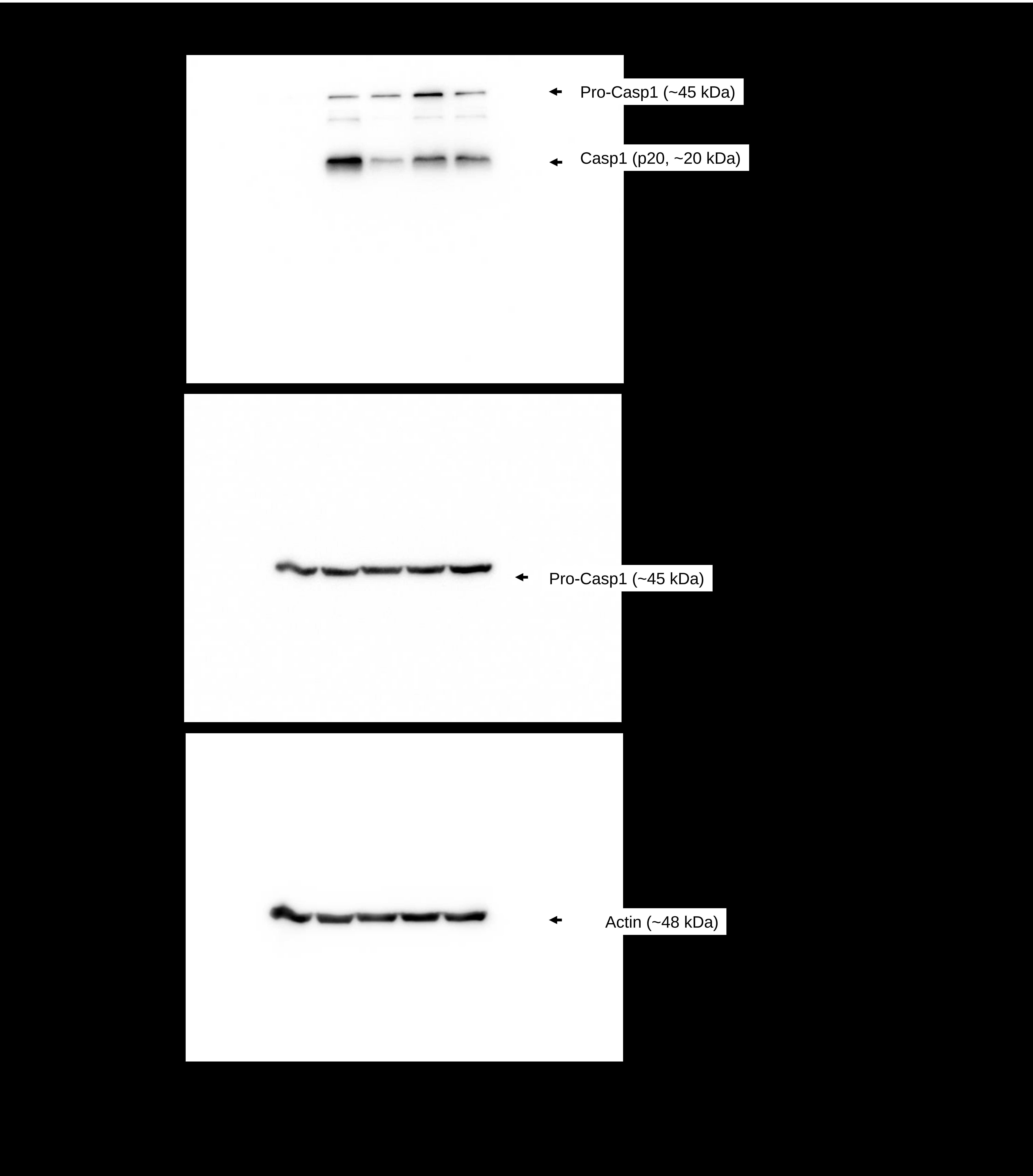

Full-length blots of Figure 5A.

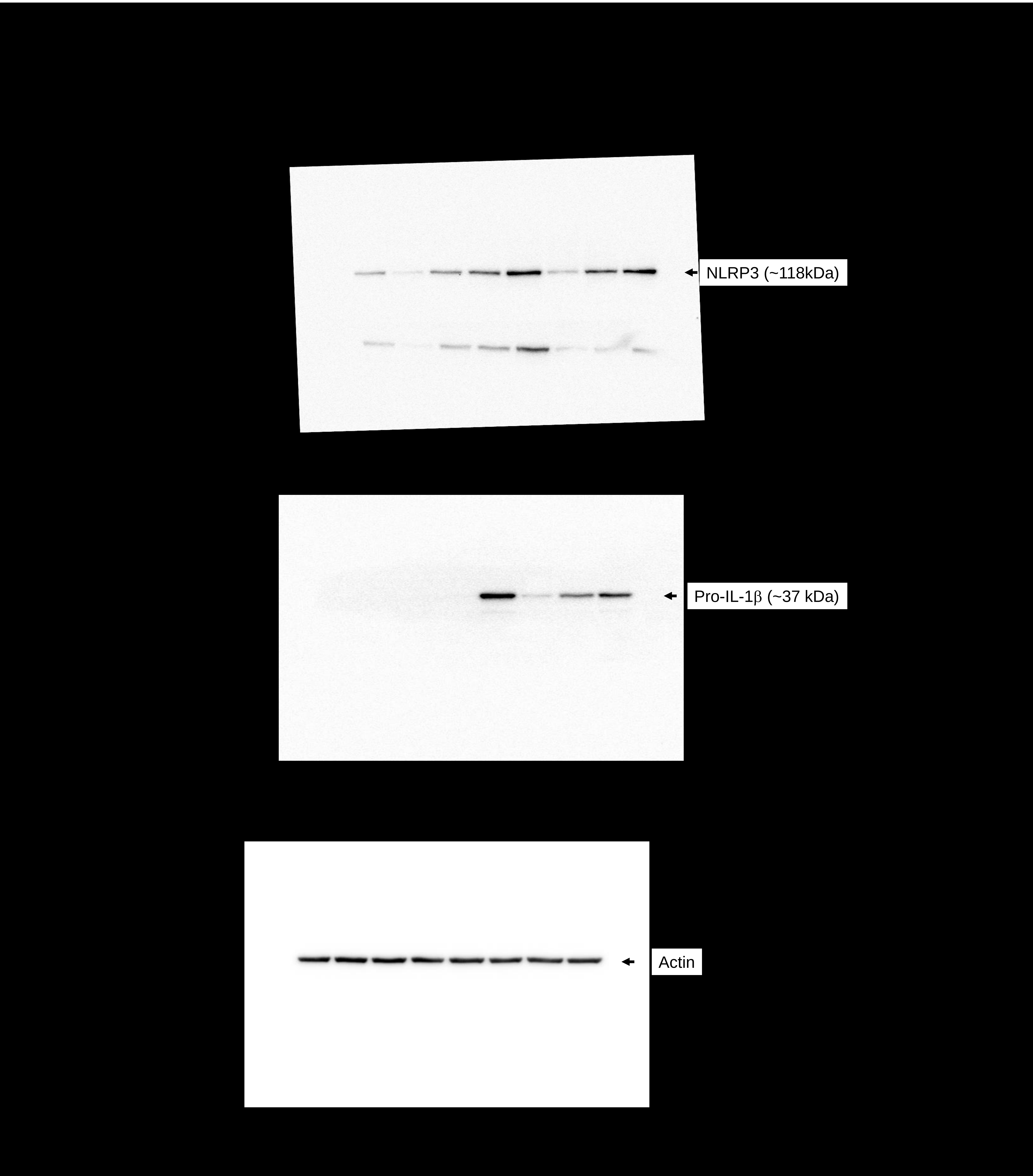

Supplemental Figure 1A

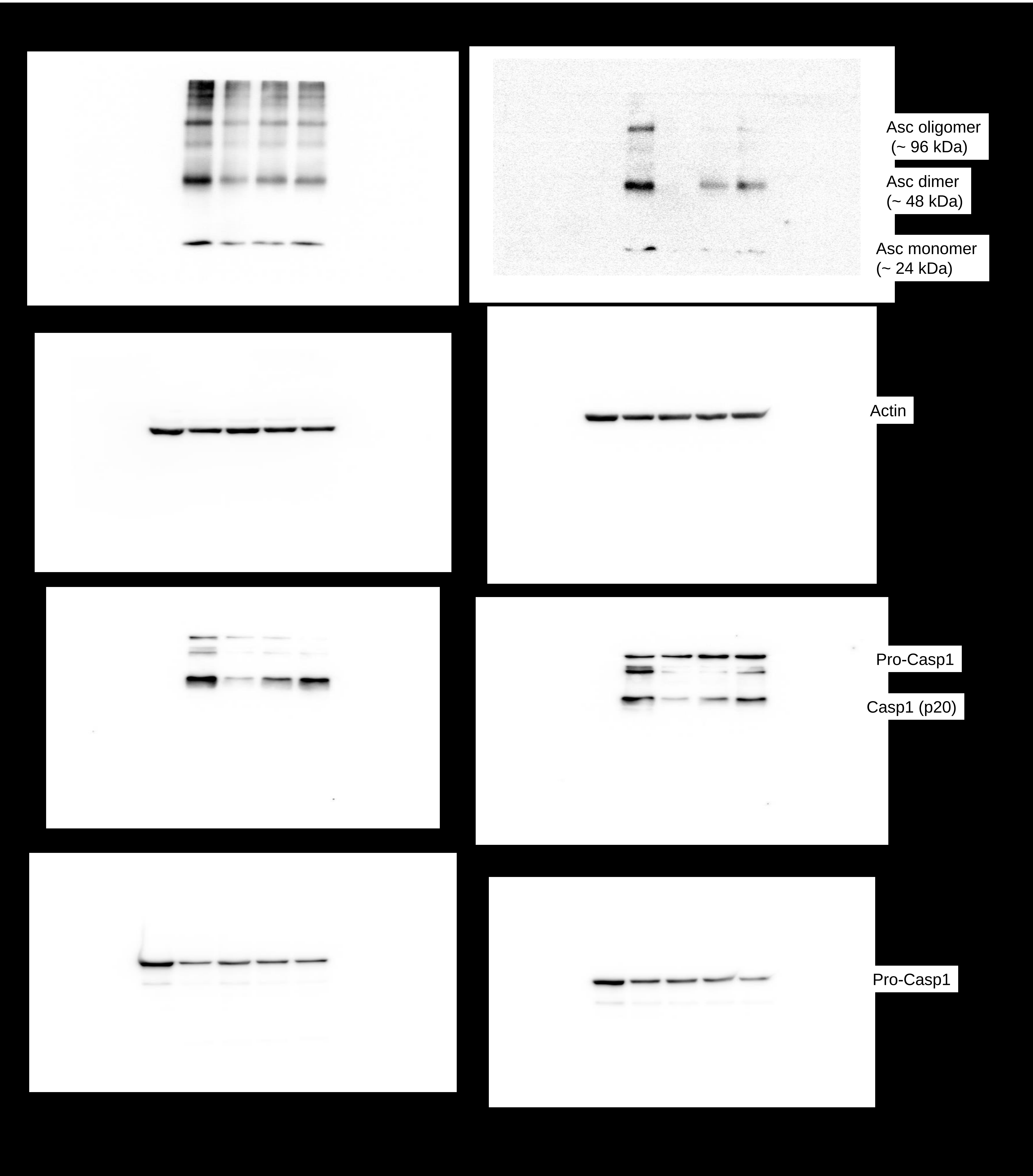

Supplemental Figure 1B

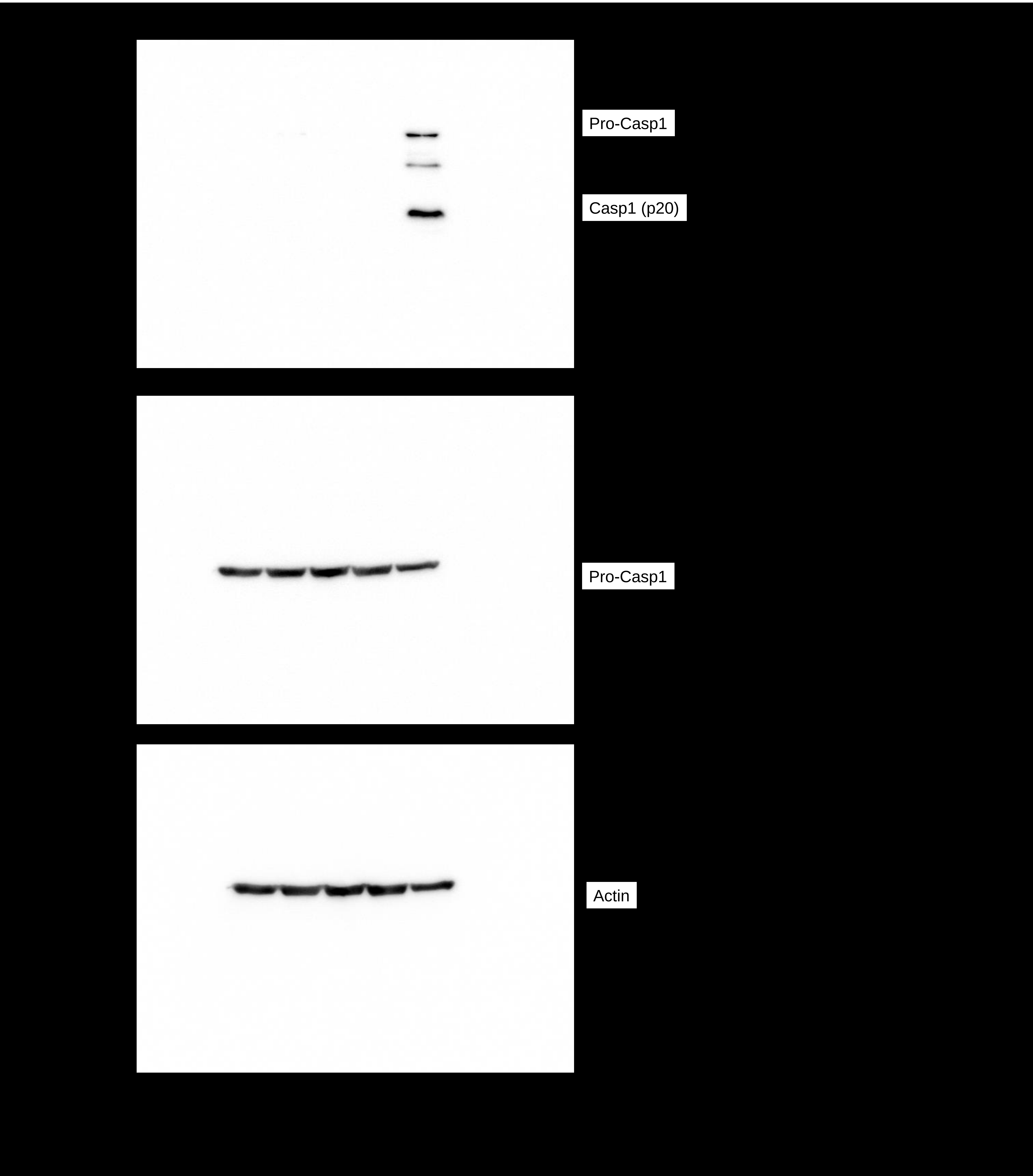

Supplemental Figure 2A

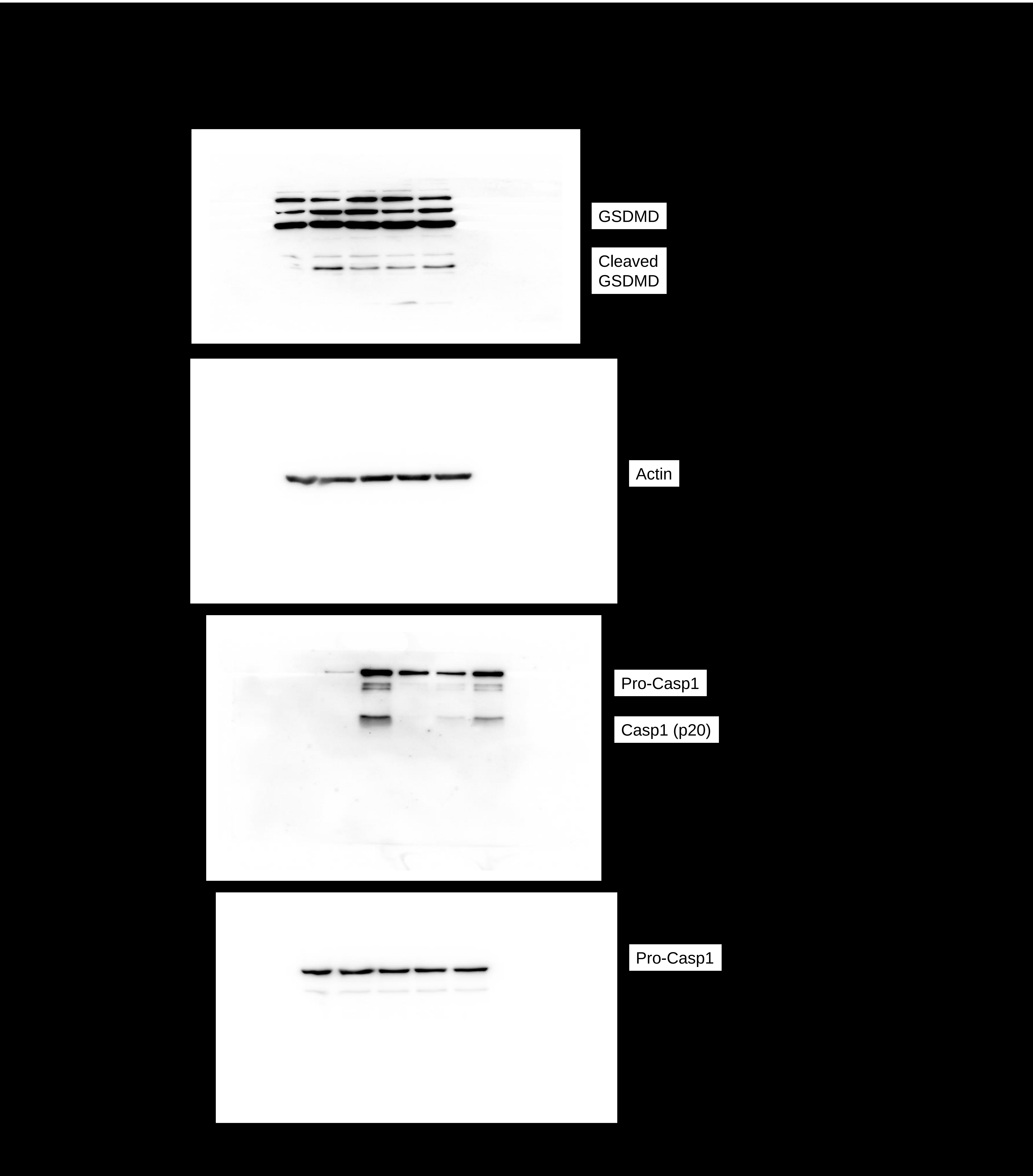

Supplemental Figure 3A

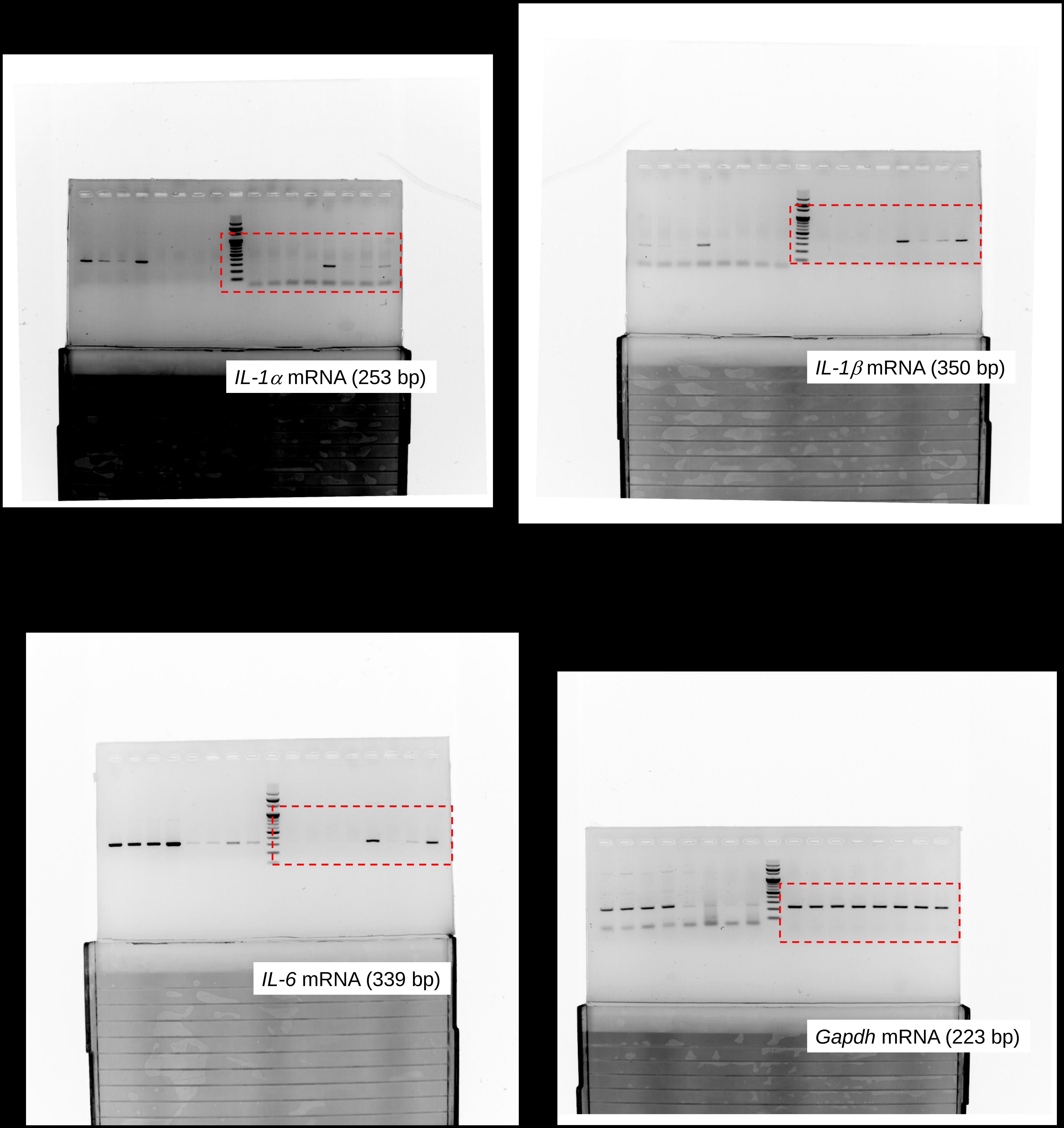

Supplemental Figure 3B

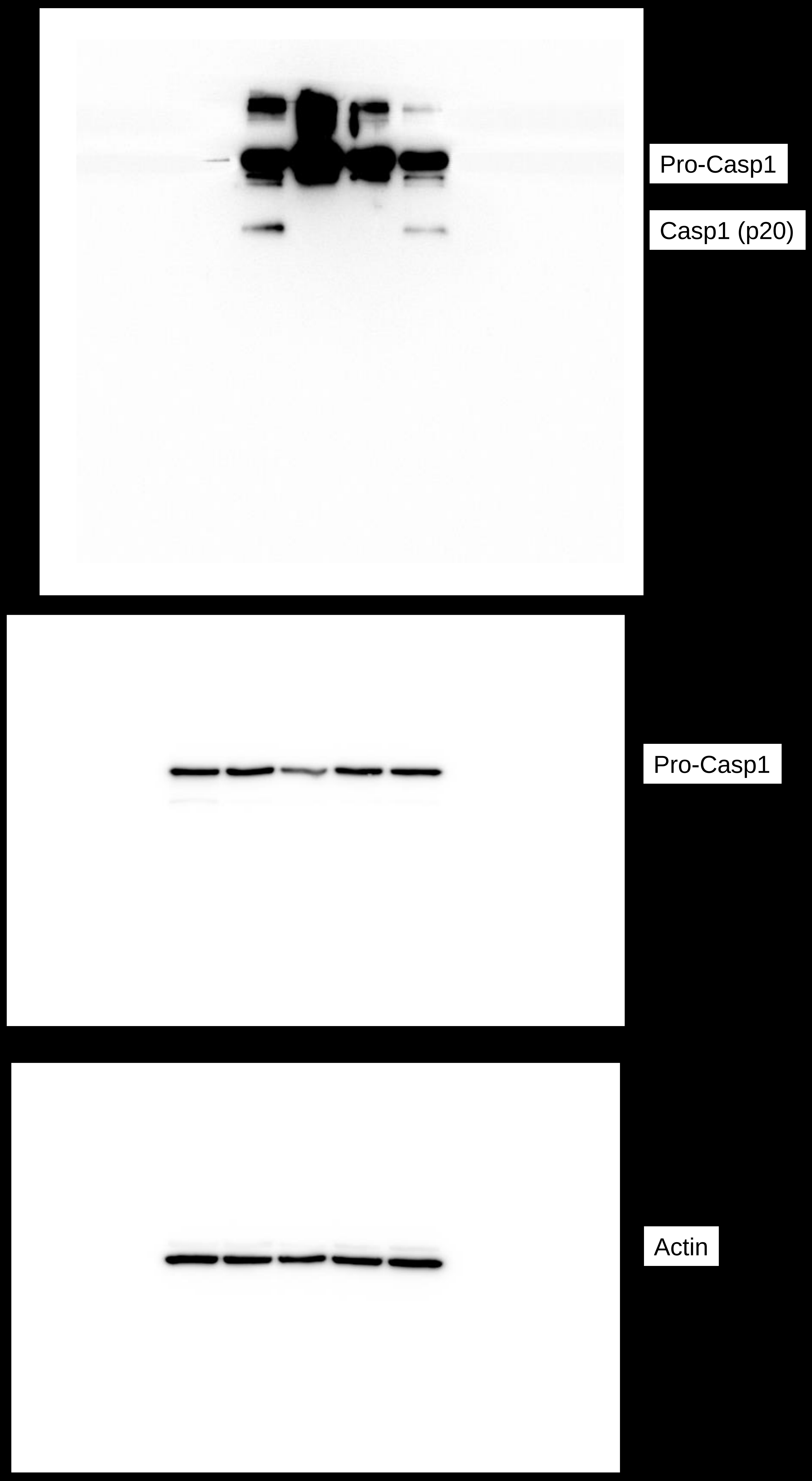

Supplement: Supplementary file 1 — Supplementary Figures. [file 41598_2022_27129_MOESM1_ESM.pdf]
